# Comparative Efficacy and Safety of Biological Agents in the Treatment of Lupus Nephritis: A Network Meta-Analysis

**DOI:** 10.1159/000527223

**Published:** 2022-11-03

**Authors:** Young Ho Lee, Gwan Gyu Song

**Affiliations:** Department of Rheumatology, Korea University College of Medicine, Seoul, Republic of Korea

**Keywords:** Biological agent, Lupus nephritis, Network meta-analysis

## Abstract

**Background:**

To date, no studies have described randomized controlled trials (RCTs) evaluating the effectiveness and safety of various biological agents used in induction therapy for lupus nephritis.

**Objectives:**

We designed this study to assess the relative efficacy and safety of some of these biological agents in patients with lupus nephritis.

**Method:**

We collected data from RCTs that examined the efficacy and safety of any biological agents for lupus nephritis and then used these data to complete a Bayesian network meta-analysis to combine the direct and indirect evidence from these studies.

**Results:**

We identified nine RCTs evaluating rituximab, abatacept, belimumab, anifrolumab, obinutuzumab, ocrelizumab, and low-dose interleukin-2 (IL-2) across 1,480 patients. Low-dose IL-2, obinutuzumab, rituximab, and belimumab achieved complete remission in a significant proportion of respondents when compared with that in the control. Ranking probability based on the surface under the cumulative ranking curve (SUCRA) indicated that low-dose IL-2 had the highest probability of achieving complete remission, followed by obinutuzumab, rituximab, belimumab, anifrolumab, abatacept, ocrelizumab, and the control. The risk of serious adverse events (SAE) tended to be lower for low-dose IL-2, rituximab, belimumab, and obinutuzumab than for the control. SUCRA-based ranking indicated that IL-2 had the highest probability of being safe, followed by rituximab, belimumab, obinutuzumab, control, anifrolumab, abatacept, and ocrelizumab.

**Conclusions:**

Low-dose IL-2 was the most effective induction treatment for patients with lupus nephritis and had the lowest potential for SAE. Higher complete remission rates and a more favorable safety profile suggest that low-dose IL-2, obinutuzumab, rituximab, and belimumab may be superior to the current control as treatments for lupus nephritis.

## Introduction

Systemic lupus erythematosus (SLE) is a systemic autoimmunity disorder with an unclear cause characterized by severe inflammation and organ damage [1]. Renal involvement occurs in up to 60% of patients with SLE, and lupus nephritis is still the leading cause of morbidity and death in SLE [2, 3]. In a 10-year follow-up, 20% of patients with lupus nephritis developed end-stage renal disease [4].

SLE is classically treated with hydroxychloroquine, glucocorticoids, methotrexate, azathioprine, mycophenolate mofetil, and cyclophosphamide (CYC) [5], all of which have been linked to the development of severe complications, including infertility, infection, and cancer, and the chances of complete remission remain low. The etiology of lupus nephritis is complicated and includes immune-mediated processes, such as autoantibody generation and immune complex deposition in the kidney [6]. However, several novel therapeutic methods have recently been developed in response to scientific breakthroughs and advances in our understanding of lupus nephritis pathophysiology. When paired with traditional medicines, many of these novel biological agents that target the lymphocytes, accessory molecules, and cytokines show improved efficacy [7]. The European Alliance of Associations for Rheumatology advises that add-on therapy with belimumab should be considered for patients with poor response to standard of care, defined as the remaining disease activity that prevents glucocorticoid tapering and/or causes repeated relapses. Rituximab may be considered for organ-threatening diseases unresponsive to traditional immunosuppressive drugs or those exhibiting intolerance/contraindications to the drugs [5]. The American College of Radiology (ACR) guideline indicates that add-on belimumab should be considered in persistently active or flare extrarenal illness, and rituximab may be used in organ-threatening and refractory disease [8]. Belimumab and anifrolumab have been approved by the FDA for the treatment of lupus nephritis.

In addition, a meta-analysis suggested that these biological therapies seem to be both safe and effective in lupus nephritis [9]. However, there is no evidence from randomized controlled trials (RCTs) comparing the relative efficacy and safety of these therapies in lupus nephritis. Thus, we designed this study to complete a network-based meta-analysis of the data to investigate the outcomes. Network meta-analyses integrate both direct and indirect evidence [10], allowing for the relative estimation of effectiveness between therapies by combining evidence from a network of RCTs, even when comparisons were not studied head-to-head [11]. Therefore, we were able to use this method to examine the efficacy and safety of these biological treatments for lupus nephritis and identify the most effective options for patients.

## Methods

### Identification of Eligible RCTs and Data Extraction

We completed an exhaustive search for studies examining the efficacy and safety of various biological agents in comparison with the control agents in patients with active lupus nephritis. This included an initial literature search performed using the MEDLINE, EMBASE, and Cochrane Controlled Trials Register databases (up to June 2022) using the following keywords and subject terms: “lupus nephritis” and “biologic agent.” All the references in each of these articles were then reviewed to identify any additional studies not included in the electronic databases. RCTs were included if they met the following inclusion criteria: (1) compared biological agent with control as induction therapy for lupus nephritis, (2) provided endpoints for the efficacy and safety of biological agents with control after induction therapy, and (3) included patients with biopsy-proven lupus nephritis. The exclusion criteria were as follows: (1) presence of duplicate data and (2) lack of adequate data for inclusion. The efficacy outcome was defined as the number of patients who achieved complete remission based on the remission criteria used in each trial. The safety outcome was defined as the number of patients who experienced serious adverse events (SAE). The following information was extracted from each study: first author, year of publication, kidney biopsy class, number of patients treated with biological agents and controls, and efficacy and safety outcomes of the drugs after induction therapy. We quantified the methodological qualities of the four studies using their Jadad scores [12]. The Jadad scale assesses random assignment, double-blinding, patient withdrawal, and dropout rates and ranges from zero to five. Quality was classified as high (score 3–5) or low (score 0–2), and network meta-analysis was conducted according to the guidelines provided by the Preferred Reporting Items for Systematic Reviews and Meta-Analyses Statement (online suppl. PRISMA Checklist; for all online suppl. material, see www.karger.com/doi/10.1159/000527223) [13].

### Evaluation of Statistical Associations for Network Meta-Analysis

The efficacy and safety of the biological agents and controls as induction therapy for lupus nephritis were ordered according to the probability of being ranked as the best performing agent. To this end, we applied a fixed-effects model and a Bayesian network meta-analysis using NetMetaXL [14] and WinBUGS statistical analysis program version 1.4.3 (MRC Biostatistics Unit, Institute of Public Health, Cambridge, UK). The Markov chain Monte Carlo method was used to obtain a pooled effect size [15], and all chains were run with 10,000 burn-in iterations followed by 10,000 monitoring iterations. The information on relative effects was converted to a probability of the treatment being the best, second best, and so on, via the ranking of each treatment using a process known as the surface under the cumulative ranking curve (SUCRA) [16] and expressed as a percentage ranging from 100% to 0% when treatment was certain to be the best and the worst, respectively. These summary estimates were then presented in league tables by ranking the treatments in the order of the most pronounced impact on the outcome under consideration based on SUCRA [16]. We then reported the pairwise odds ratio and 95% credible interval adjusted for multiple-arm trials, and these pooled results were considered significant if the 95% credible interval did not contain a value of 1.

### Inconsistency and Sensitivity Analyses

Inconsistency refers to the extent of disagreement between the direct and indirect evidence [17], and its assessment is critical when conducting a network meta-analysis [18]. Given this, we plotted the posterior mean deviance of the individual data points in the inconsistency model against their posterior mean deviance in the consistency model to assess network inconsistency between the direct and indirect estimates in each loop [19]. We then completed a sensitivity test by comparing fixed- and random-effects models to ensure our analysis was both sensitive and consistent.

## Results

### Studies Included in the Meta-Analysis

Our evaluations identified a total of 995 studies, 14 of which were selected for a full-text review based on their title and abstract details. However, five studies were excluded because they contained non-RCT data, no outcome data, or review data. Thus, nine RCTs comprising 1,504 patients met the inclusion criteria [20, 21, 22, 23, 24, 25, 26, 27, 28]. There were two studies each on rituximab and abatacept and one study each on belimumab, obinutuzumab, anifrolumab, ocrelizumab, and low-dose IL-2 (Table 1). Most of the patients were in their 30s. Ethnicities included one European, two Asian, and six mixed ethnic groups. All patients received standard of care as background therapy that could have contributed to the results (Table 1). The Jadad scores for all except one of these studies ranged from 3 to 4, indicating high study quality (Table 1). The relevant features of these studies are presented in Table 1.

### Network Meta-Analysis of Biological Agent Efficacy

**Fig. 1:**
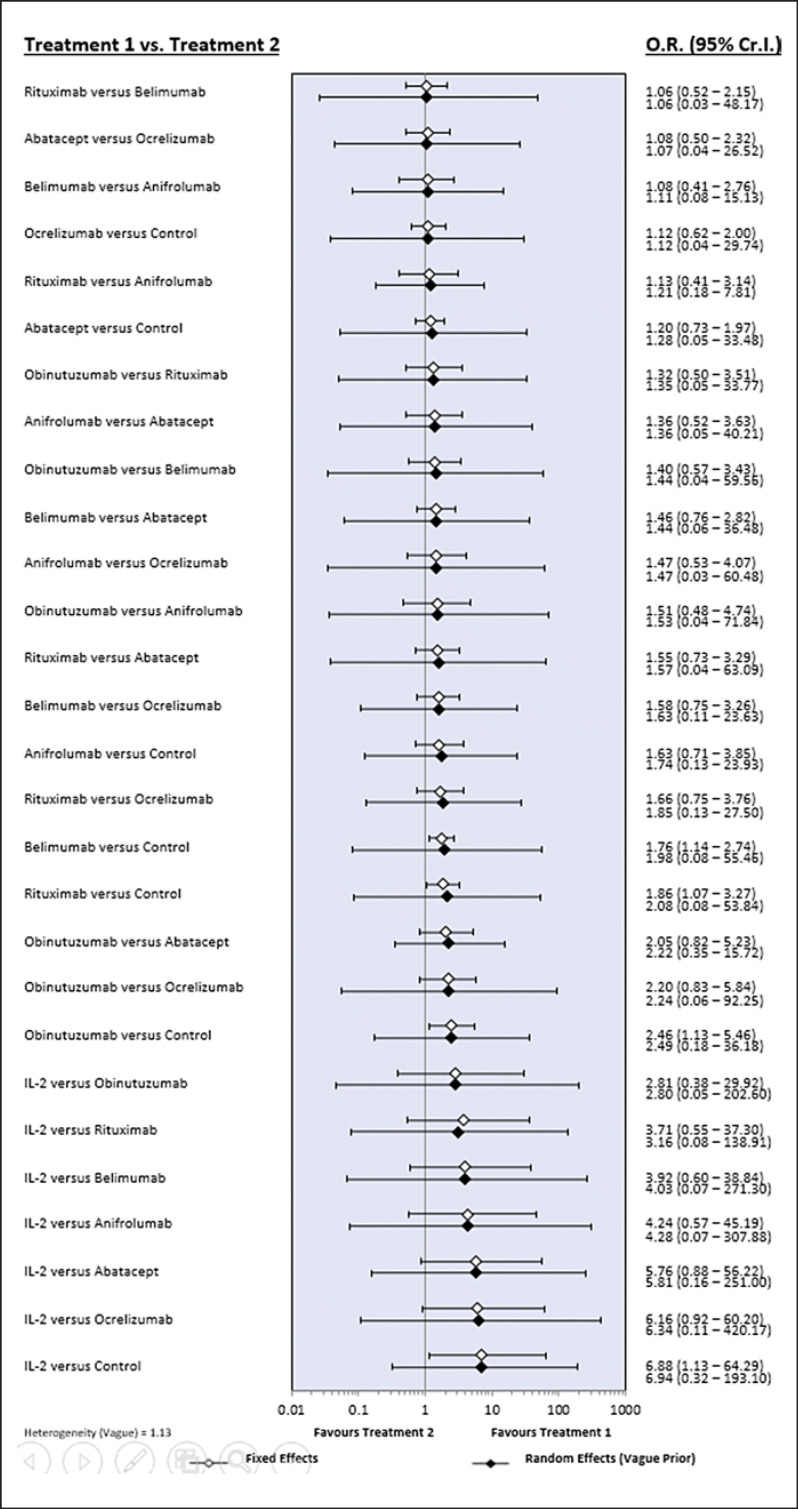
Bayesian network meta-analysis results of various randomized controlled studies evaluating the relative efficacy of biological agents for complete remission in lupus nephritis.

Our evaluations used the number of complete remissions as the direct measure of efficacy. Thus, low-dose IL-2 is listed diagonally in the top left and bottom right of the league tables as it demonstrated both the most and least favorable SUCRA results, respectively (Tables 2, 3). In addition, our evaluations revealed that compared with the control, low-dose IL-2, obinutuzumab, rituximab, and belimumab are likely to achieve a significant complete remission response (Table 2; Fig. 1). Ranking probability based on SUCRA indicates that low-dose IL-2 has the highest probability of being the best treatment for achieving complete remission, followed by obinutuzumab, rituximab, belimumab, anifrolumab, abatacept, ocrelizumab, and the control (Table 4).

### Network Meta-Analysis of the Safety of Biological Agents

**Fig. 2:**
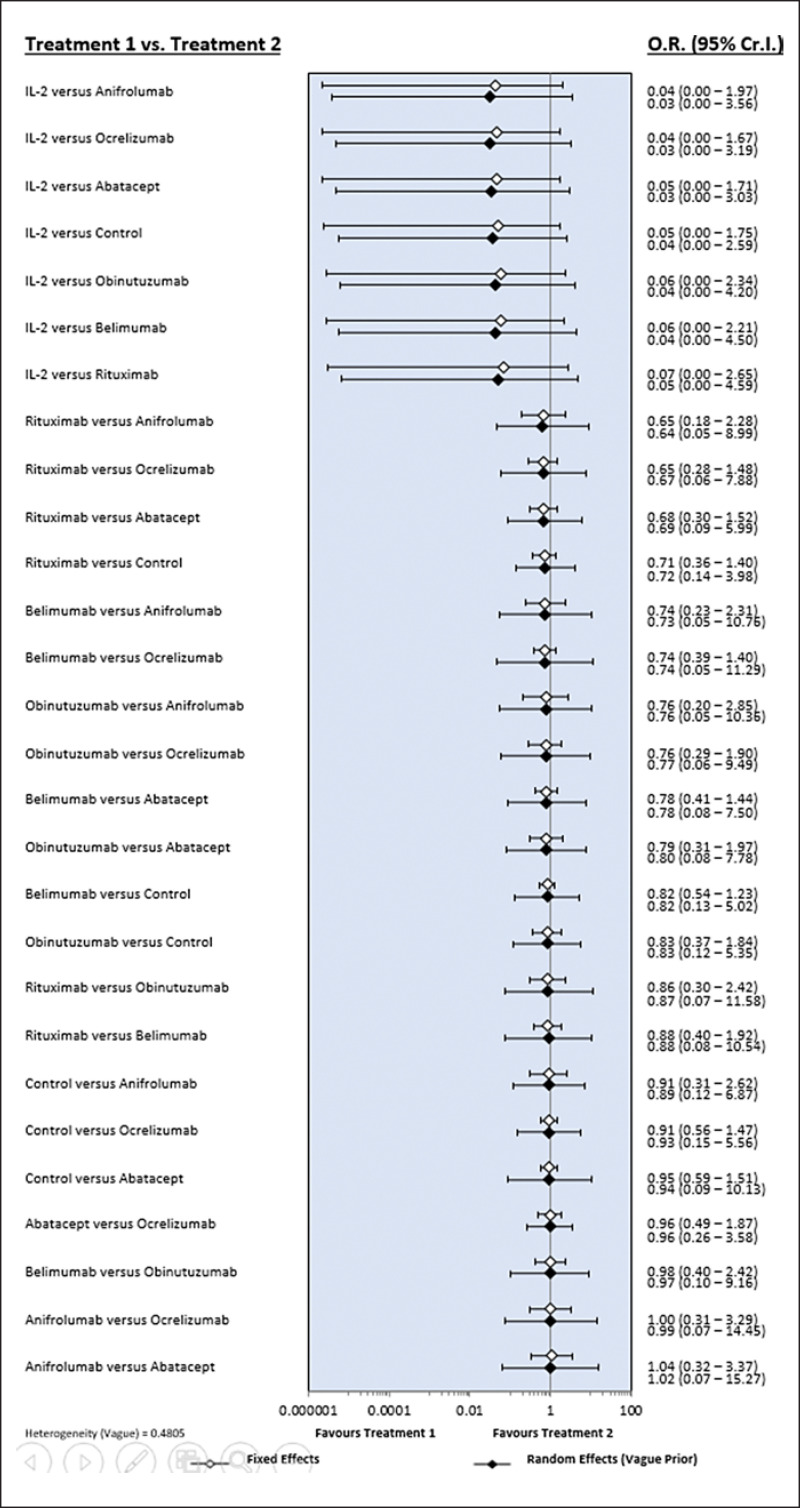
Bayesian network meta-analysis results for various randomized controlled studies describing the relative safety and incidence of serious adverse reactions in response to different biological agents during the treatment of lupus nephritis.

The risk of SAE was significantly lower with low-dose IL-2 than with the control, ocrelizumab, and anifrolumab (Table 3; Fig. 2), with SUCRA-based evaluations suggesting that low-dose IL-2 had the highest probability of being the safest treatment, followed by rituximab, belimumab, obinutuzumab, abatacept, control, ocrelizumab, and anifrolumab (Table 4).

### Inconsistency and Sensitivity Analyses

Inconsistency plots assessing network inconsistencies between the direct and indirect estimates revealed a low possibility of inconsistencies affecting the network meta-analysis results. In addition, the results of the random- and fixed-effects models provided the same interpretation, indicating that the results of this network meta-analysis are robust (Fig. 1, 2).

## Discussion

Here, we collected and analyzed the available data to determine the relative efficacy and safety of various biological agents used in the treatment of patients with lupus nephritis. Our data showed that low-dose IL-2 was the most effective therapy for lupus nephritis, followed by obinutuzumab, rituximab, and belimumab, all of which outperformed the control. Low-dose IL-2 had the lowest risk of SAE and was the safest medicine, followed by rituximab, belimumab, and obinutuzumab.

Treatment with IL-2 may boost virus-specific CD8+ T-cell responses and stimulate natural killer cell activity against infection [29]. In patients with lupus nephritis, low-dose IL-2 therapy resulted in complete remission in 53.85% (7/13) of cases compared with only 16.67% (2/12) in the placebo group (*p* = 0.036) [26]. Obinutuzumab is a humanized type II anti-CD20 monoclonal antibody that binds to the CD20 antigen and is glycoengineered for increased affinity toward FcRIII on effector cells [27]. Following 104 weeks of observation, obinutuzumab achieved a higher rate of complete response (26 [41%] vs. 14 [23%], *p* = 0.026) than the traditional control, indicating its improved efficacy. Rituximab, a chimeric monoclonal antibody, targets CD20+ B cells without affecting the stem and plasma cells [30]. A recent study showed that rituximab increased the total (complete and partial) therapeutic response rates of 72 patients to 56.9% when compared with only 45.8% for the control (*p* = 0.18). Another study showed that the total response rate of rituximab was 83.3%, which was substantially greater than that of CYC at only 57.1% (*p* < 0.05) [24]. Belimumab, a recombinant human IgG1 monoclonal antibody that suppresses B-cell-activating factor, was approved for use in patients with active autoantibody-positive SLE and was recently shown to increase the response rate in patients by up to 10% when compared with that for the control (30% vs. 20%, *p* = 0.02) [25].

Previous meta-analyses found that, compared with control agents, biological agents were safe to use, with no additional adverse outcomes [9]. This meta-analysis varied from that conducted by Chen et al. as we added a new RCT on anifrolumab [28] but excluded the trial on atacicept [31] since it was discontinued owing to the accompanied unexpected, significant, and rapid decreases in IgG levels, as well as high incidence of serious infections. Unlike standard meta-analyses, we used network meta-analysis to rank the effectiveness and safety of several biological treatments by pooling direct and indirect data to obtain the overall estimates [15, 32, 33].

Despite the clear outcomes, our results should be interpreted with caution owing to the various limitations of our study. First, only a few RCTs with limited sample sizes were included. Each biological drug had one or two studies, and the number of patients with SLE in three studies is <100. The less number of patients with SLE administered these therapies and included in the network meta-analysis might hinder a precise fulfillment of the study's objectives and affect the accuracy of the results by skewing them. Second, the variability in the design and patient characteristics of the included studies may have impacted the results of this network meta-analysis. For example, the response to immunosuppressive medications used to treat lupus nephritis varies according to ethnicity. All patients received standard of care − combinations of glucocorticoid with immunosuppressive agents, such as CYC or mycophenolate mofetil. Third, this study did not fully address the efficacy and safety outcomes of biological drugs as it focused primarily on the number of patients who had a complete response or showed SAE rather than examining a variety of outcomes. Nonetheless, there were some benefits to this meta-analysis. We ranked the effectiveness and safety of biologics for the treatment of lupus nephritis. The number of people with lupus nephritis in the included studies varied from 25 to 446, but this pooled study included 1,480 people. Unlike individual studies, we could provide more exact data by increasing the statistical power and precision by merging the results of many analyses [34, 35].

In conclusion, using a Bayesian network meta-analysis encompassing nine RCTs, we found that low-dose IL-2 was the most effective biological therapy for patients with lupus nephritis and had the best chance of lowering the risk of SAE. Higher complete remission rates along with a more favorable safety profile indicate that low-dose IL-2, obinutuzumab, rituximab, and belimumab are better therapies for lupus nephritis than other treatments. However, further research is required to definitively assess the relative effectiveness and safety of these biological medicines in a large patient cohort.

## Statement of Ethics

An ethics statement is not applicable because this study is based exclusively on published literature.

## Conflict of Interest Statement

The authors have no conflicts of interest to declare.

## Funding Sources

There were no funding sources.

## Author Contributions

Young Ho Lee was involved in conception and design of study, acquisition of data, analysis and/or interpretation of data, drafting the manuscript, and revising the manuscript critically for important intellectual content. Gwan Gyu Song was involved in conception and acquisition of data, analysis and/or interpretation of data, and revising the manuscript critically for important intellectual content.

## Data Availability Statement

All data generated or analyzed during this study are included in this article. Further inquiries can be directed to the corresponding author.

## Supplementary Material

Supplementary data

## Figures and Tables

**A:** 

Study [Ref.]	Ethnicity	N biopsy class	Age, years mean ± SD	Total number	Treatment	Standard of care	Patients, *n*	Follow-up period
Furie, 2022 [27]	Mixed^a^	III, IV, V	33.1±9.8	125	Obinutuzumab	MMF	63	104 weeks
					Control		62	

Jayne, 2022 [28]	European	III, IV, V	35.0 (18–65)^b^	132	Anifrolumab	MMF	87	52 weeks
					Control		45	

Furie, 2020 [25]	Mixed^c^	III, IV, V	33.7±10.7	446	Belimumab	CYC or MMF	223	104 weeks
					Control		223	

He, 2020 [26]	Asian	Biopsy-proven LN	31.58±9.25	25	Low-dose IL-2	CYC, MMF, or other immunosuppressants^d^	13	24 weeks
					Control		12	

Zhang, 2015 [24]		III, IV, V			Rituximab	CYC	42	12 months
					Control		42	

ACCESS, 2014 [22]	Mixed^e^	III, IV, V	32.0±10.1	134	Abatacept	CYC	66	24 weeks
					Control		68	

Furie, 2014 [22]	Mixed^f^	III, IV, V	31.0±9.5	199	Abatacept	MMF	99	52 weeks
					Control		100	

Mysler, 2013 [21]	Mixed^g^	III, IV, V	31.3 (16–69)^b^	223	Ocrelizumab	CYC or MMF	148	48 weeks
					Control		75	

Rovin, 2012 [20]	Mixed^h^	III, IV, V	31.8±9.6	144	Rituximab	MMF	72	78 weeks
					Control		72	

**B:** 

Study [Ref.]		Name		Mechanism		Dosage of biological agents		Jadad score
Furie, 2022 [27]		Obinutuzumab		A humanized type II anti-CD20 monoclonal antibody		Obinutuzumab was administered as a blinded intravenous infusion of 1,000 mg on day 1 and weeks 2, 24, and 26		4

Jayne, 2022 [28]		Anifrolumab		Type I interferon receptor antibody		900 mg for the first three doses, 300 mg thereafter intravenously every 4 weeks for 48 weeks		3

Furie, 2020 [25]		Belimumab		A recombinant human IgG-1λ monoclonal antibody that inhibits B-cell-activating factor		Patients received intravenous belimumab at a dose of 10 mg per kilogram of body weight		4

He, 2020 [26]		Low-dose IL-2		Expansion of immune tolerance-inducing Treg cells and suppression of effector T cells		IL-2 (1 million IU) was administered subcutaneously every other day for 2 weeks (seven injections), followed by a 2-week break, as one treatment cycle of 4 weeks		3

Zhang, 2015 [24]		Rituximab		A chimeric monoclonal antibody that depletes CD20+ B cells		Patients received intravenous pulse dose of 375 2 mg/m^2^ at weeks 0, 2, 4, and 6		2

ACCESS, 2014 [23]		Abatacept		A fusion protein comprising CTLA-4 linked to the Fc portion of IgG1		Treatment was initiated with monthly infusions of abatacept at doses (for <60 kg, 500 mg; for 60–100 kg, 750 mg; for 100 kg, 1 gm)		3

Furie, 2014 [22]		Abatacept		A fusion protein comprising CTLA-4 linked to the Fc portion of IgG1		Patients received abatacept 30 mg/kg on days 1, 15, 29, and 57, followed by abatacept approximating 10 mg/kg (weight tiered: 500 mg for patients weighing <60 kg, 750 mg for patients 60–100 kg, 1,000 mg for patients 100 kg) on days 85, 113, 141, 169, 197, 225, 253, 281, 309, and 337		3

Mysler, 2013 [21]		Ocrelizumab		A recombinant humanized monoclonal antibody that depletes CD20 B cells		Patients received 400 mg or 1,000 mg ocrelizumab given as an intravenous infusion on days 1 and 15, followed by a single infusion at week 16 and every 16 weeks thereafter		3

Rovin, 2012 [20]		Rituximab		A chimeric monoclonal antibody that depletes CD20+ B cells		Patients received rituximab 1,000 mg administered intravenously on days 1, 15, 168, and 182		3

**C:** 

Comparison	Study number	Patient number						
Control	9	703						
Abatacept	2	165						
Obinutuzumab	1	63						
Belimumab	1	223						
IL-2	1	13						
Ocrelizumab	1	148						
Rituximab	2	114						
Anifrolumab	1	51						

LN, lupus nephritis; SD, standard deviation; IL-2, interleukin-2; CTLA-4, cytotoxic T-lymphocyte antigen 4; CYC, cyclophosphamide; MMF, mycophenolate mofetil.

a Latin America and Caribbean 60%, Europe and Israel 29%, the USA 11%.

b Range, mixed.

c Asian 223 (50%), white 148 (33%), black 61 (14%), American Indian or Alaska Native 10 (2%), and multiple races 4 (1%).

d Azathioprine, cyclosporine, tacrolimus, leflunomide, thalidomide, or methotrexate.

e White 51%, African American 41%, Asian 3%, undeclared 3%.

f Asian 60.6%, white 28.3%, African American 6.1%, other 5.1%.

g Latin America 42%, Asia 23.1%, Western Europe 12.9%, Eastern Europe 10.5%, the USA and Canada 10.2%, Africa 1.3%.

h White 26.4%, black 27.8%, Hispanic 40.3%, Asian/Pacific Islander 5.6%.

**Table 2:** League tables showing the results of the network meta-analysis comparing the effects of all drugs including odds ratios and 95% credible intervals

IL-2					
2.81 (0.38–29.92)	Obinutuzumab					
3.71 (0.55–37.30)	1.32 (0.50–3.51)	Rituximab				
3.92 (0.60–38.84)	1.40 (0.57–3.43)	1.06 (0.52–2.15)	Belimumab			
4.24 (0.57–45.19)	1.51 (0.48–4.74)	1.13 (0.41–3.14)	1.08 (0.41–2.76)	Anifrolumab		
5.76 (0.88–56.22)	2.05 (0.82–5.23)	1.55 (0.73–3.29)	1.46 (0.76–2.82)	1.36 (0.52–3.63)	Abatacept	
6.16 (0.92–60.20)	2.20 (0.83–5.84)	1.66 (0.75–3.76)	1.58 (0.75–3.26)	1.47 (0.53–4.07)	1.08 (0.50–2.32)	Ocrelizumab
6.88 (1.13–64.29)	2.46 (1.13–5.46)	1.86 (1.07–3.27)	1.76 (1.14–2.74)	1.63 (0.71–3.85)	1.20 (0.73–1.97)	1.12 (0.62–2.00) Control

**Table 3:** League tables showing the results of the network meta-analysis comparing the effects of all drugs including odds ratios and 95% credible intervals

IL-2						
0.07 (0.00–2.65)	Rituximab						
0.06 (0.00–2.21)	0.88 (0.40–1.92)	Belimumab					
0.06 (0.00–2.34)	0.86 (0.30–2.42)	0.98 (0.40–2.42)	Obinutuzumab				
0.05 (0.00–1.75)	0.71 (0.36–1.40)	0.82 (0.54–1.23)	0.83 (0.37–1.84)	Control			
0.04 (0.00–1.97)	0.65 (0.18–2.28)	0.74 (0.23–2.31)	0.76 (0.20–2.85)	0.91 (0.31–2.62)	Anifrolumab		
0.05 (0.00–1.71)	0.68 (0.30–1.52)	0.78 (0.41–1.44)	0.79 (0.31–1.97)	0.95 (0.59–1.51)	1.04 (0.32–3.37)	Abatacept	
0.04 (0.00–1.67)	0.65 (0.28–1.48)	0.74 (0.39–1.40)	0.76 (0.29–1.90)	0.91 (0.56–1.47)	1.00 (0.31–3.29)	0.96 (0.49–1.87)	Ocrelizumab

**Table 4:** Rank probability of the efficacy of biologic agents based on the number of patients who achieved a complete remission (A) response and the safety based on the number of SAE (B)

Treatment	SUCRA
A. Efficacy	
IL-2	0.929
Obinutuzumab	0.754
Rituximab	0.612
Belimumab	0.581
Anifrolumab	0.505
Abatacept	0.280
Ocrelizumab	0.230
Control	0.110
B. Safety	
IL-2	0.931
Rituximab	0.657
Belimumab	0.587
Obinutuzumab	0.529
Control	0.361
Anifrolumab	0.335
Abatacept	0.319
Ocrelizumab	0.282

SUCRA, surface under the cumulative ranking curve.
